# Circ-10720 as a ceRNA adsorbs microRNA-1238 and modulates ZEB2 to boost NSCLC development by activating EMT

**DOI:** 10.1186/s40001-024-01715-9

**Published:** 2024-04-12

**Authors:** Wei Zhang, Ping Xiao, Bin Liu, Yan Zhang

**Affiliations:** 1 Department of Medical Oncology, Sichuan Cancer Hospital & Institute, Sichuan Cancer Center, School of Medicine, University of Electronic Science and Technology of China, People’s South Road, Section 4, Number 55, Chengdu, 610041 Sichuan China; 2 Department of Thoracic Surgery, Sichuan Cancer Hospital & Institute, Sichuan Cancer Center, School of Medicine, University of Electronic Science and Technology of China, Chengdu, 610041 Sichuan China; 3 Department of Medical Oncology, Cancer Center, West China Hospital, Sichuan University, 37 Guoxue Lane, Wuhou District, Chengdu, 610041 Sichuan China; 4 Lung Cancer Center/Lung Cancer Institute, West China Hospital, Sichuan University, Chengdu, 610041 Sichuan China

**Keywords:** Circular RNA-10720, MicroRNA-1238, Zinc Finger E-box-binding Homeobox 2, Non-small cell lung cancer, Epithelial–mesenchymal transition

## Abstract

**Background:**

Circular RNAs (circRNAs) are critical regulators in the progression of tumors. This experimental design aimed to explore the mechanism of circ-10720 in non-small cell lung cancer (NSCLC).

**Methods:**

We used RT-qPCR to measure circ-10720 expression in clinical samples and analyzed its relationship with the clinicopathological characteristics of NSCLC patients. The expression levels of microRNA-1238 (miR-1238) and Zinc Finger E-box-binding Homeobox 2 (ZEB2) in clinical samples were detected by RT-qPCR. NSCLC cells were transfected with relevant plasmids or sequences. Circ-10720, miR-1238, and ZEB2 expressions in cells were analyzed via RT-qPCR or western blot. Cell proliferation, apoptosis, migration, and invasion were assessed with CCK-8, flow cytometry, and transwell assay, respectively. The protein expression of ZEB2 and epithelial–mesenchymal transition (EMT)-related markers (E-cadherin, Vimentin, N-cadherin) were detected via western blot. Xenograft assay was used to determine the effect of circ-10720 on NSCLC in vivo. Circ-10720 and ZEB2 expressions in tumors were detected using RT-qPCR or Western blot. Immunohistochemistry was used to evaluate E-cadherin and N-cadherin expression in tumors. Finally, the binding relationship between miR-1238 with circ-10720 or ZEB2 was verified by the bioinformatics website, dual luciferase reporter assay, RNA pull-down assay, and RIP assay.

**Results:**

Circ-10720 was upregulated in NSCLC and correlated with TNM stage of NSCLC patients. MiR-1238 was lowly expressed but ZEB2 was highly expressed in NSCLC. Circ-10720 silencing suppressed the proliferation, metastasis, and EMT of NSCLC cells. Mechanically, circ-10720 was a competitive endogenous RNA (ceRNA) for miR-1238, and ZEB2 was a target of miR-1238. circ-10720-modulated ZEB2 via competitively binding with miR-1238 to control NSCLC progression. In addition, circ-10720 knockdown suppressed tumor growth in vivo.

**Conclusions:**

Circ-10720 acts as a ceRNA to adsorb miR-1238 and modulate ZEB2 to facilitate the proliferation, migration, invasion, and EMT of NSCLC cells.

**Graphical Abstract:**

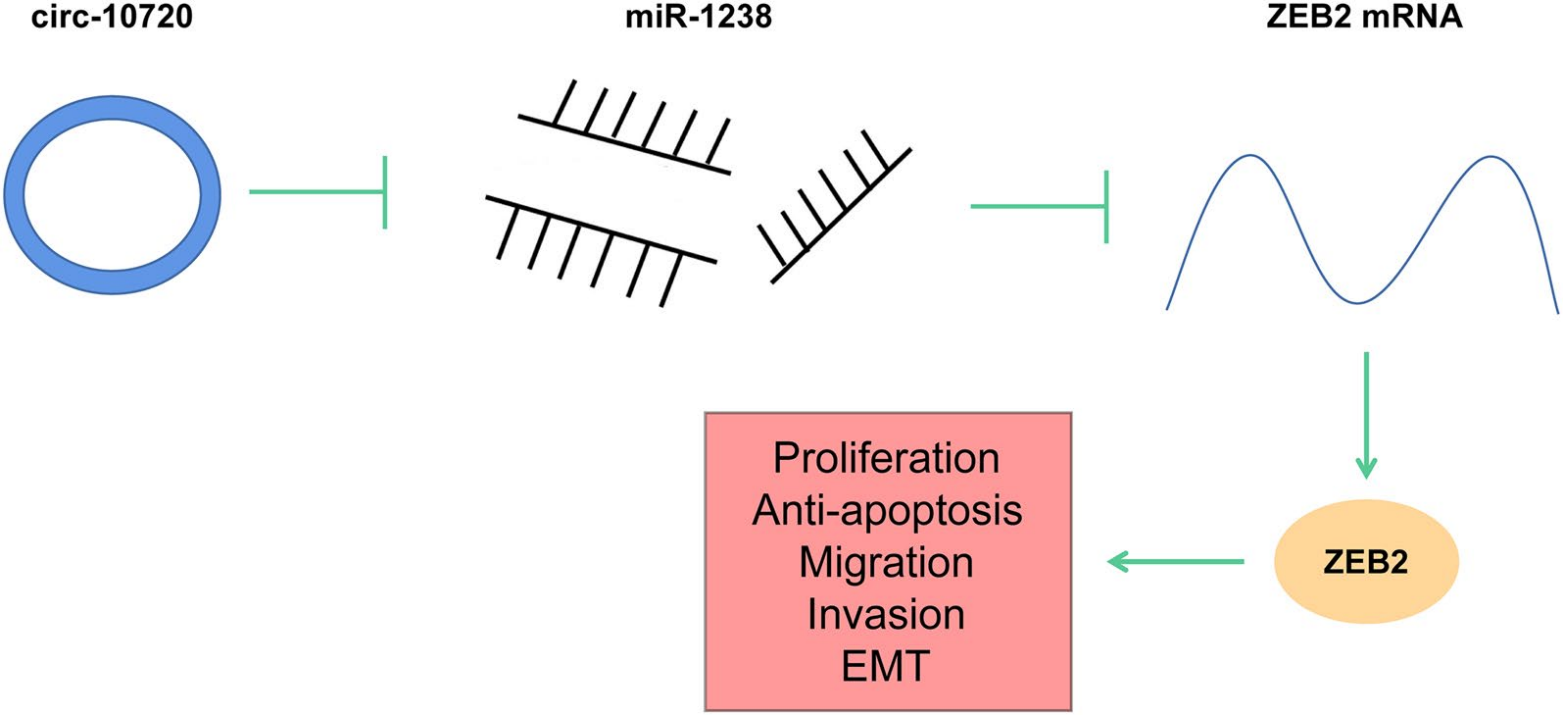

## Introduction

Lung cancer is the most prevalent cancer [1]. The mortality of lung cancer has decreased slightly over the past few years, but the number of deaths in 2017 still exceeded that of prostate, breast, brain, and colorectal cancers combined [2]. The global morbidity and mortality of lung cancer have come up to 11.6% and 18.4%, respectively [3]. Lung cancer is divided primarily into small-cell lung cancer (SCLC) and non-small cell lung cancer (NSCLC). NSCLC makes up over 80% of all lung cancer cases [4]. Tremendous advances have been made in chemotherapy, surgical treatment, and molecular targeted therapy, but the prognosis for patients with NSCLC is yet poor, and the 5-year survival rate of the patient is scarcely about 15% of patients with advanced NSCLC [5]. Consequently, it is urgent to figure out the pathogenesis of NSCLC and develop novel treatments and methods to enhance the survival rate.

Circular RNA (circRNA) is a non-coding RNA molecule with a closed loop structure and without 5ʹ-cap and 3ʹ-poly (A) structures [6]. CircRNAs are widely present in various eukaryotes and have many biological characteristics [7], and they can serve as miRNA sponges, protein scaffolds, protein decoys and even have a translational function [8–10]. circRNA exerts a vital part in the occurrence and development of human diseases, particularly cancer [11]. Increasing evidence has indicated that differential expression of circRNAs in tissues or blood shows a certain correlation in the early diagnosis and prognosis evaluation of lung cancer, and is expected to become a potential biomarker of lung cancer [12, 13]. In addition, recent studies have highlighted the association between circRNAs and the occurrence and development of lung cancer, especially with lung cancer metastasis [14]. CircPTK2 represses TGF-β-stimulated EMT and metastasis in NSCLC via modulating TIF1γ [15]. CircSWT1 promotes the invasion, migration, and EMT of NSCLC cells via the miR-370-3p/SNAIL axis [16]. Epithelial–mesenchymal transition (EMT) is a reversible developmental genetic process involving the transformation of polarized epithelial cells into mesenchymal cells [17]. EMT exerts a critical part in tumor metastasis and has drawn attention for its function in the resistance of traditional and targeted therapies [18]. During EMT, cells lose epithelial characteristics, parietal polarity, cytoskeletal structure, and intercellular adhesion complexes characteristic of epithelial tissues, while gaining mesenchymal cell polarity, independent migration and invasive capacity [19]. The acknowledged markers of EMT are (E-cadherin) and vimentin [20]. Repression of EMT is supposed to be a promising treatment strategy for NSCLC.

A novel circRNA named circ-10720 (CircBase ID: hsa_circ_0018189), which is derived from the CUL gene in stem cell carcinoma, can modulate NSCLC cell progression and EMT and performs as a biomarker for NSCLC recurrence [21]. circ-10720 has been verified to be a carcinogenic factor in hepatocellular carcinoma. circ-10720 can serve as a competitive endogenous RNA (ceRNA) for miR-490-5p, miR-578, and miR-1246 to upregulate VIM protein levels, thus activating a mesenchymal phenotype [22, 23]. However, more studies are required to explore the role and molecular mechanism of circ-10720 in NSCLC*.*

CircRNAs have been well-acknowledged to function as miRNA sponge as well as transcription regulators to regulate gene expressions. This study was to figure out its mechanism in NSCLC. According to our preliminary results from bioinformatic analysis, both circ-10720 and ZEB2 contain potential binding sites for miR-1238. Therefore, we propose the hypothesis that circ-10720 acts as a ceRNA to adsorb miR-1238 and regulate ZEB2 to promote EMT-mediated NSCLC progression.

## Materials and methods

### Research objects

The collected NSCLC endoscopic biopsy specimens were confirmed by histopathology in Sichuan Cancer Hospital, including 96 NSCLC specimens and 96 paracancerous tissue specimens. Inclusion criteria: all patients were pathologically diagnosed with NSCLC [24]; the patients had no history of radiotherapy or chemotherapy. Exclusion criteria: metastatic lung cancer; long-term administration of glucocorticoid drugs; combined systemic infectious diseases and other diseases that influence this study. All patients signed informed consent for this study, and authorization was obtained from the Ethics Committee of Sichuan Cancer Hospital and Institute.

### Cell culture

Normal bronchial epithelial cell line 16HBE and human NSCLC cell lines (H1299, HCC44, A549, and A549) were purchased from the Chinese Academy of Sciences (Shanghai Institute of Cell Biology, Shanghai, China). A complete culture medium containing 10% fetal bovine serum (FBS) was used, and different culture medium met different cell culture requirements. RMP11640, MEM, and Dulbecco’s Modified Eagle Medium (DMEM) were used (all HyClone Biological Company, USA).

### RNase R treatment

The stability of circ-10720 was detected [25]. RNase R (5 U/µg, Epicentre; Illumina, Inc.) was added to 2 mg total RNA separated from A549 or HCC44 cells and incubated for 30 min. Subsequently, RNAs was purified using RNeasy MinElute Cleaning kit (Qiagen, Inc.). circRNA or mRNA expression was analyzed.

### Cell transfection

Cells (2 × 10^5^ cells/well) were seeded into 6-well cell culture plates 36 h prior to transfection. Cells were incubated with a serum-free medium for 1 h until cell confluency reached 60%. Transfection of 50 nM si-NC, si-circ-10720, mimic-NC, miR-1238 mimic, pcDNA3.1-ZEB2, and miR-1238 inhibitor was done in line with the instructions of Lipofectamine 2000 transfection reagent (Invitrogen, USA). All plasmids and sequences were purchased (all Shanghai GenePharma Co. Ltd., Shanghai, China), and specific sequences were not disclosed for commercial reasons.

### Cell counting kit (CCK)-8 assay

Cells were digested into a single-cell suspension with 0.25% trypsin according to the instructions of the CCK-8 kit (Beijing Sora Bio). Cells were seeded in a 96-well plate with a cell density of 0.5 × 10^4^, and 100 μL of cell suspension was prepared (100 μL/well, about 5000 cells). The plates were sealed with PBS solution and pre-incubated in an incubator that met the experimental requirements. Samples were taken at 24, 48, and 72 h after the cells adhered to the wall, and 10 μL of CCK-8 solution was added to each well and incubated in the dark for 2 h. The optical density value (OD) was measured by a microplate reader (BD Company, USA). The cell proliferation curve was drawn, with time as the abscissa and OD as the ordinate [26]

### Flow cytometry

After trypsin digestion and centrifugation, cells were tested by Annexin V-fluorescein isothiocyanate (FITC)/propidium iodide (PI) apoptosis detection Kit (all BD Company). Cells were suspended in 400 μL of 1 × AnnexinV-binding solution, and the cell density was adjusted to 1 × 10^6^/mL. Then, 5 μL of Annexin V-FITC staining solution was added and incubated in the dark for 15 min. In addition, 10 μL of PI staining solution was added, and cells were tested using a flow cytometer (BD company, model: FACSCalibur). Cells were divided into 4 quadrants with fluorescein isothiocyanate (FITC) rabbit anti-mouse antibody and PI fluorescence as a two-parameter dot plot: viable cells in the lower left quadrant, early apoptotic cells in the lower right quadrant, late apoptotic cells and dead cells in the upper right quadrant, and mechanically damaged cells in the upper left quadrant. Apoptosis rate = early apoptosis + late apoptosis [27]

### Transwell

Transfected cells were obtained in each group, and the cell concentration was adjusted to 2.0 × 10^5^/mL. A total of 200 μL of cell suspension was added to Matrigel diluted with serum-free cell culture medium to coat the upper chamber of Transwell (no Matrigel in the migration test), and 600 μL of DMEM was added to cover the lower Transwell chamber containing 10% FBS. After 24 h, cells were taken and fixed in paraformaldehyde for 20 min. The number of transmembrane cells in 6 fields of view was randomly counted under the microscope, and the average value was taken [28].

### RNA extraction and reverse transcription-quantitative polymerase chain reaction (RT-qPCR)

Tissues and cells were collected and added with TRIZOL lysis buffer to extract total RNA, and the concentration and purity were determined using a microplate reader. Reverse transcription of the extracted RNA was performed using a reverse transcription kit (all Dalian TaKaRa Company), and PCR amplification reaction was performed. Glyceraldehyde-3-phosphate dehydrogenase (GAPDH) was the internal control for circ-10720 and ZEB2, and U6 was that for miR-1238. PCR primers were purchased (Shanghai Genechem Co., Ltd., Shanghai, China). circ-10720, forward: 5ʹ-GTCGTATCCAGTGCAGGG-3ʹ, reverse: 5ʹ-CGACGCTTCCTCGTCTG-3ʹ; miR-1238, forward: 5ʹ-GCGCTTCCTCGTCTGTC-3ʹ, reverse: 5ʹ-GCAGGGTCCGAGGTATTC-3ʹ; ZEB2, forward: 5ʹ-AGCGACACGGCCATTATTTAC-3ʹ, reverse: 5ʹ-GTTGGGCAAAAGCATCTGGAG-3ʹ; U6, forward: 5ʹ-CTCGCTTCGGCAGCACA-3ʹ, reverse: 5ʹ-AACGCTTCACGAATTTGCGT-3ʹ; GAPDH, forward: 5ʹ-CACCCACTCCTCCACCTTTG-3ʹ, reverse: 5ʹ-CCACCACCCTGTTGCTGTAG-3ʹ. PCR products were electrophoresed on a 2% agarose gel, and the results were scanned and analyzed using a gel imaging analysis system. The 2^−ΔΔCt^ method was used to calculate target genes [29].

### Western blot

Tissues and cells were collected, lysed with radio-immunoprecipitation assay cell lysis and protease inhibitor on ice, and centrifuged to take the supernatant. Determination of the protein concentration was done in line with the instructions of the BCA kit. Total protein (80 μL) was separated by 12% sodium dodecyl sulfate gel electrophoresis and transferred to polyvinylidene fluoride membranes. After treatment with 5% skimmed milk powder for 2 h, the primary antibodies ZEB2 (1:1000), E-cadherin (1:1000), Vimentin (1:2000), N-cadherin (1:1000), GAPDH (1:10,000) (Abcam, Cambridge, MA, USA) were added. After incubation with goat anti-rabbit Immunoglobulin G (IgG) or goat anti-mouse IgG (1: 2000), the bands were visualized by enhanced chemiluminescence [30].

### Fluorescence in situ hybridization (FISH)

FISH was performed using a fluorescence in situ hybridization kit (Promega, Beijing, China). Specific probes for circ-10720 (cy3-labeled) and miR-1238 (FAM-labeled) (Servicebio, Wuhan, China) were used for in situ hybridization, and nuclei were simultaneously stained by DAPI. Images were taken using an Eclipse Ti-sr microscope (Nikon, Japan) [31].

### The luciferase reporter experiment

Bioinformatics website predicted the sequence of circ-10720 or ZEB2 3′-untranslated region (UTR) binding to miR-1238. After amplification, circ-10720 or ZEB2 3ʹ-UTR mutant sequences were obtained using a site-directed mutagenesis kit (National Bureau of Statistics, Beijing). The amplified circ-10720 or ZEB2 3ʹ-UTR sequences and circ-10720 or ZEB2 3ʹ-UTR mutant sequences were inserted into the psi-CHECK2 reporter plasmid (Promega, USA). After sequencing analysis, WT-circ-10720 or WT-ZEB2 3ʹ-UTR plasmid and Mutant-circ-10720 or Mutant-ZEB2 3ʹ-UTR plasmid were obtained. According to the instructions of Lipofectamine™ 2000 transfection reagent, miR-1238 mimic or negative control was transfected. Samples were collected 48 h after transfection, and the relative luciferase activity assay was conducted using the dual luciferase assay kit (Promega, Shanghai) [32].

### RNA-pull down

Biotin-labeled miR-1238 wild-type plasmid and the biotin-labeled miR-1238 mutant plasmid (50 nM each) were transfected into the cells, respectively. After transfection of 48 h, cells were incubated with a specific cell lysis buffer (Ambion, Austin, Texas, USA) for 10 min and with M-280 streptavidin magnetic beads (Sigma, St. Louis, MO, USA) pre-coated with RNase-free and yeast tRNA (Sigma, St. Louis, MO, USA) for 3 h. Then, the sample was eluted, total RNA was extracted via Trizol, and detection of circ-10720 was performed [33].

### RNA immunoprecipitation (RIP)

An assessment was performed using the Magna RIP RNA-Binding Protein Immunoprecipitation Kit (Millipore, Bedford, MA, USA). Cells were lysed with a complete RIP lysis buffer and incubated with magnetic beads, which were conjugated with human anti-Argonaute2 (Ago2) antibody or normal mouse IgG (Millipore). After 24 h, the magnetic beads were treated with Proteinase K to digest protein. Ultimately, an analysis of the immunoprecipitated RNA was performed [34].

### Nude mouse tumor xenograft experiment

In this experiment, 20 nude mice (male, 4 weeks old) (both Beijing Huafukang Biotechnology Co., Ltd., Beijing, China) were selected and raised in sterile laminar flow animal rooms (SPF grade). Lentivirus plasmids of short hairpin RNA (shRNA) against circ-10720 (sh-circ-10720) and negative control (sh-NC) were constructed by Genepharma (Shanghai, China). Cells were infected with lentiviral particles of sh-circ-10720 or sh-NC, and then, nude mice were injected with 2 × 10^6^ cells into the subcutaneous area of the right back. The date and time of cell inoculation were recorded, and each nude mouse was marked. After 7 days, the longest diameter (length) and shortest diameter (width) of the tumors were measured with a ruler sterilized by UV radiation. After that, tumor volume was calculated: *π*/6 × (length × width)^2^. After 4 weeks, mouse euthanasia was done with CO_2_, and tumors were weighed [35].

### Immunohistochemistry

The tumor tissue was taken, fixed with 4% paraformaldehyde solution, dehydrated, and sliced into 4 μm. Sections were routinely dewaxed and hydrated and added with H_2_O_2_, and microwaves were used to retrieve antigens. The sample was blocked with 10% normal goat serum, and E-cadherin (1:200, American Abgent Company) and N-cadherin (1:200, American Affinity Company) antibodies were added. After the addition of a secondary antibody (Proteintech Company, USA), the sections were developed with diaminobenzidine, followed by staining with hematoxylin and sealing with neutral resin. The stained sections showed brown–yellow granules under the microscope. Ten fields were randomly selected for photography, and analysis of the images was done with Image J software (Media Cybernetics) [36].

### Statistical analysis

Analysis of the data was done using GraphPad Prism 8. The measurement data were shown in the form of mean ± standard deviation (SD). The comparison between two groups was performed by *t* test. The comparison among multiple groups was done using one-way analysis of variance (ANOVA) and Tukey’s multiple comparisons test. The correlation between circ-10720 and clinicopathological characteristics of patients was determined by Chi-square test. *P* < 0.05 was accepted as indicative of distinct differences.

## Results

### Circ-10720 expression is augmented in NSCLC and is correlated with the patient’s TNM stage and disease severity

**Fig. 1:**
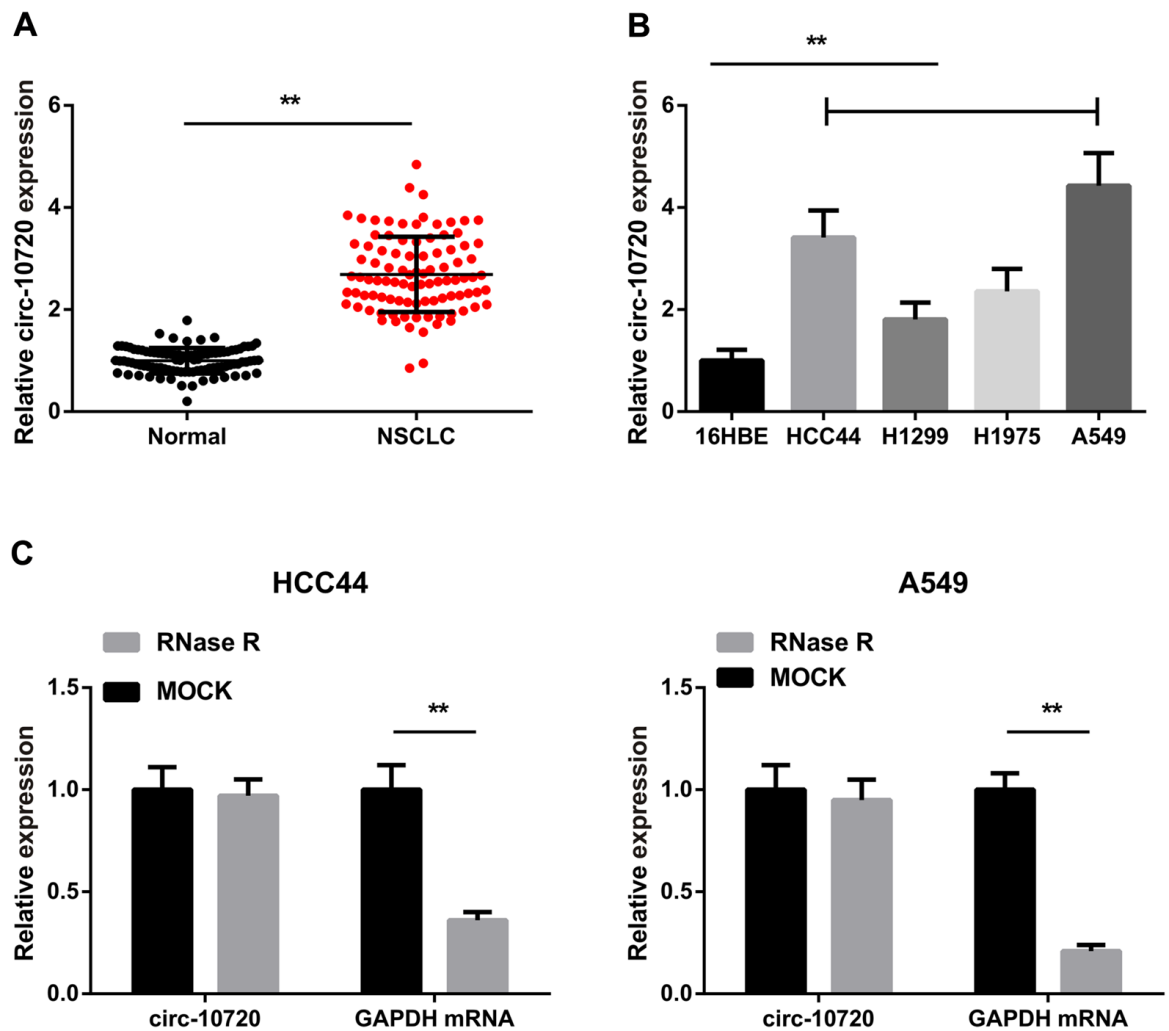
Circ-10720 is elevated in NSCLC and is correlated with patients’ clinical severity. **A** Test of circ-10720 in NSCLC tissues and para-cancerous tissues by RT-qPCR; **B** detection of circ-10720 in NSCLC cell lines HCC44, H1299, H1975, A549 and Normal bronchial epithelial cell line 16HBE by RT-qPCR; **C** RNase R treatment to testify the circular features of circ-10720; *P < 0.05, **P < 0.01. The data in the figure were in the form of mean ± SD; the correlation between circ-10720 and clinicopathological features of patients was analyzed by Chi-square test

Examination of circ-10720 in NSCLC tissues and corresponding para-cancerous tissues was conducted, which elucidated that circ-10720 expression was augmented versus the para-cancerous tissues (Fig. 1A). Then, we divided NSCLC patients into two groups based on the median expression of circ-10720 (Low expression group, *n* = 48; high expression group, *n* = 48). As shown in Table 1, TNM stage grade (*P* = 0.006), differentiation (*P* = 0.035) and tumor size (*P* = 0.008) rather than age and gender were significantly correlated with high levels of circ-10720 expression in NSCLC.

**Table 1:** Association of circ-10720 with pathological parameters of NSCLC

Clinical indexes	*n*	Circ-10720		*P*
		The declined (48)	The elevated (48)	
Age				0.525
60 or less	61 (63.5%)	29 (47.5%)	32 (52.5%)	
More than 60	35 (36.5%)	19 (54.3%)	16 (45.7%)	
Gender				0.146
Male	57 (59.4%)	25 (43.9%)	32 (56.1%)	
Female	39 (40.6%)	23 (59.0%)	16 (41.0%)	
Differentiation				0.035
Well and moderate	36 (37.5%)	23 (63.9%)	13 (36.1%)	
Poor	60 (62.5%)	25(41.7%)	35 (58.3%)	
Tumor size				0.008
5 or less	53 (55.2%)	33 (62.3%)	20 (37.7%)	
More than 5	43 (44.8%)	15 (34.9%)	28 (65.1%)	
Tumor stage				0.006
I–II	61 (63.5%)	37 (60.7%)	24 (29.3%)	
III–IV	35 (36.5%)	11 (31.4%)	24 (68.6%)	

Circ-10720 expression was also elevated in NSCLC cell lines, particularly in A549 and HCC44 cells (Fig. 1B). In addition, compared with GAPDH treated with RNase R, the stability assay of circ-10720 was more stable, indicating that circ-10720 has a ring structure (Fig. 1C).

### Circ-10720 accelerates cell malignant behavior of NSCLC via regulating miR-1238

**Fig. 2:**
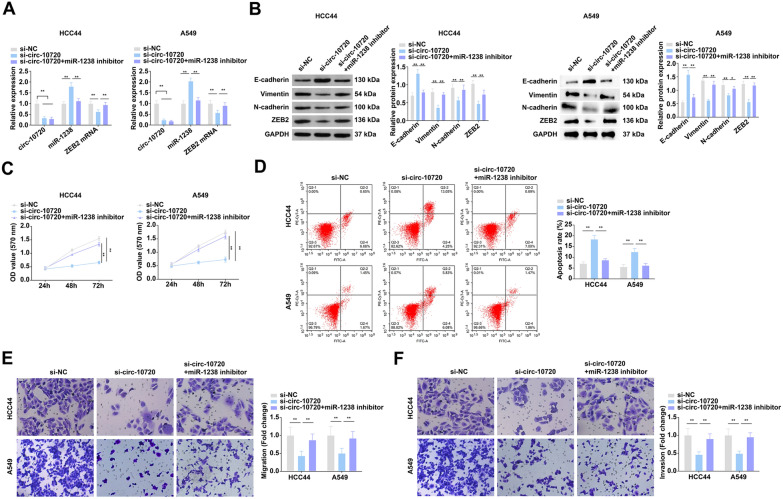
Circ-10720 accelerates cell malignant behavior of NSCLC via regulating miR-1238. **A** Examination of circ-10720, miR-1238, and ZEB2 mRNA in cells after transfection by RT-qPCR; **B** detection of ZEB2, E-cadherin, N-cadherin, and Vimentin in cells by Western blot; **C** test of cell proliferation after transfection by CCK8 assay; **D** detection of cell apoptosis rate after transfection by flow cytometry; **E**, **F** test of cell migration and invasion after transfection by Transwell; *P < 0.05, **P < 0.01. *N* = 3; the data in the figure were in the form of mean ± SD

RT-qPCR and Western blot were adopted to test the expression levels of circ-10720, miR-1238, and ZEB2 in A549 and HCC44 cells after si-circ-10720 transfection. The results displayed that si-circ-10720 successfully knocked down circ-10720 in cells, while elevating miR-1238 expression and reducing ZEB2 expression (Fig. 2A, B).

Examination of EMT-correlated proteins E-cadherin, Vimentin, and N-cadherin was performed (Fig. 2B). After knocking down circ-10720, E-cadherin protein was elevated, and N-cadherin and Vimentin were reduced. Cell progression was determined, clarifying that knockdown of circ-10720 restrained the cell development (Fig. 2C–F). Moreover, to investigate whether circ-10720 regulates miR-1238-mediated progression of NSCLC cells, miR-1238 expression was inhibited in cells transfected with si-circ-10720 using miR-1238 inhibitor. It was manifested that miR-1238 inhibitor effectively turned around the influence of knocking down circ-10720 on NSCLC cells. The above results indicated that circ-10720 accelerated cell malignant behavior of NSCLC via modulating miR-1238.

### MiR-1238 attenuates NSCLC cell growth, metastasis, and EMT by targeting ZEB2

**Fig. 3:**
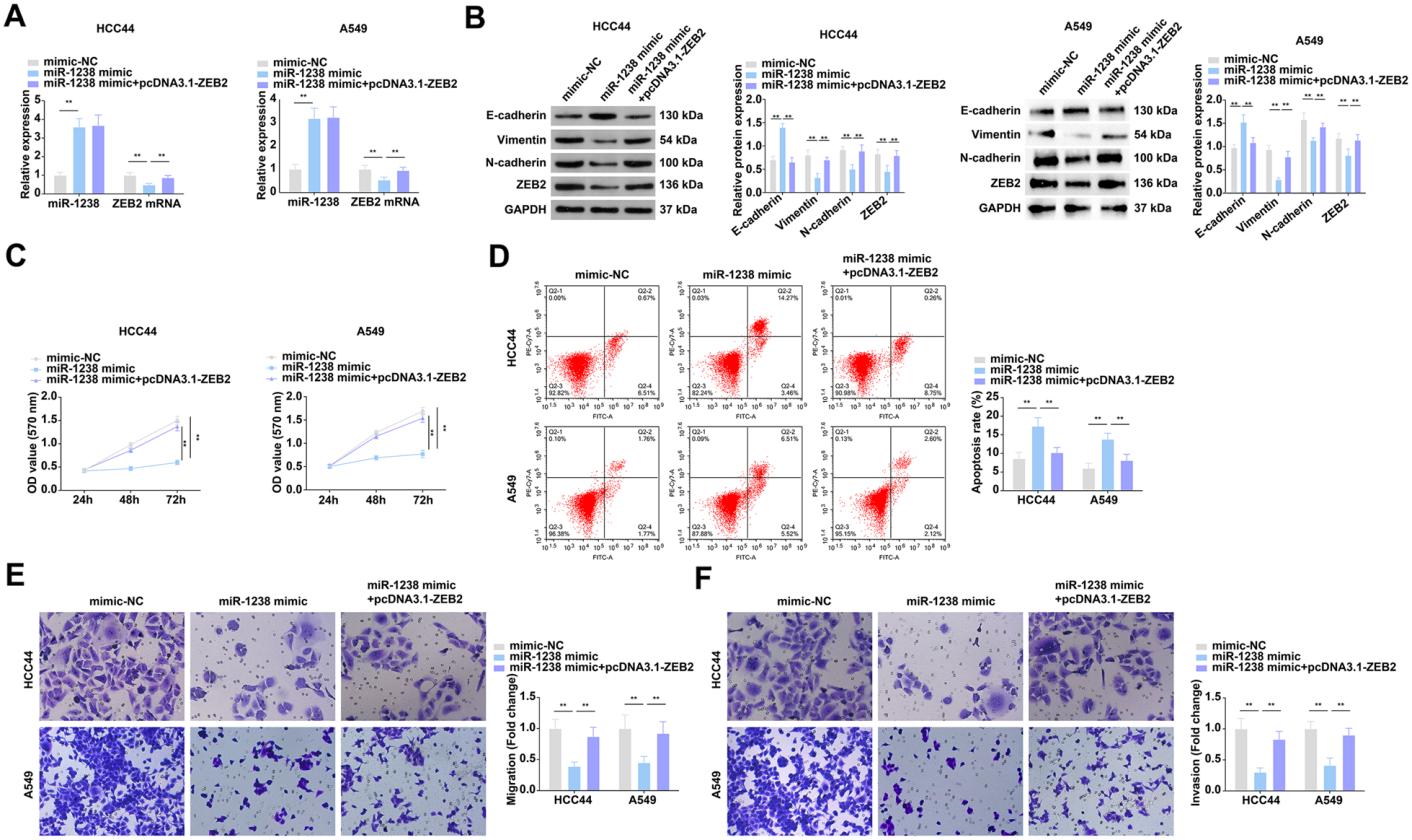
MiR-1238 attenuates NSCLC cell growth, metastasis, and EMT by targeting ZEB2. **A** Test of miR-1238 and ZEB2 mRNA in cells after transfection by RT-qPCR; **B** examination of cell ZEB2 and E-cadherin, N-cadherin, Vimentin by Western blot; **C** test of cell proliferation after transfection by CCK8 assay; **D** detection of cell apoptosis rate after transfection by flow cytometry; **E**, **F** examination of cell migration and invasion after transfection by Transwell; *P < 0.05, ** *P* < 0.01. *N* = 3; the data in the figure were in the form of mean ± SD

To explore the effects of miR-1238 on NSCLC cells, miR-1238 mimic was transfected. The transfection efficiency of miR-1238 mimic was checked, and cells after transfection demonstrated reduction in ZEB2 expression (Fig. 3A, B).

Western blot results found that miR-1238 mimic could enhance the expression of E-cadherin, retard the expression of N-cadherin and Vimentin (Fig. 3B). Elevation of miR-1238 repressed the development of NSCLC cells (Fig. 3C–F). Moreover, transfection of pcDNA3.1-ZEB2 on the basis of miR-1238 mimic turned around the influence of miR-1238 mimic on NSCLC cell biology. These observations indicated that miR-1238 attenuates NSCLC cell growth, metastasis, and EMT by targeting ZEB2.

### Circ-10720 modulates ZEB2 via sponge adsorption of miR-1238

MiR-1238 is reported as a suppressor in multiple cancers, such as prostate cancer [37, 38], colorectal cancer [39], etc. A recent study has shown that circKIF4A sponges miR-1238 to promote NSCLC progression by up-regulating claudin14 (CLDN14) expression [40]. In addition to that, Shi et al. found that miR-1238 inhibits tumor cell proliferation of NSCLC by targeting LHX2 [41]. According to our previous preliminary results from the bioinformatic analysis, both circ-10720 and ZEB2 contain potential binding sites for miR-1238. Therefore, the potential circ-10720/miR-1238/ZEB2 axis might be involved in the progression of NSCLC.

**Fig. 4:**
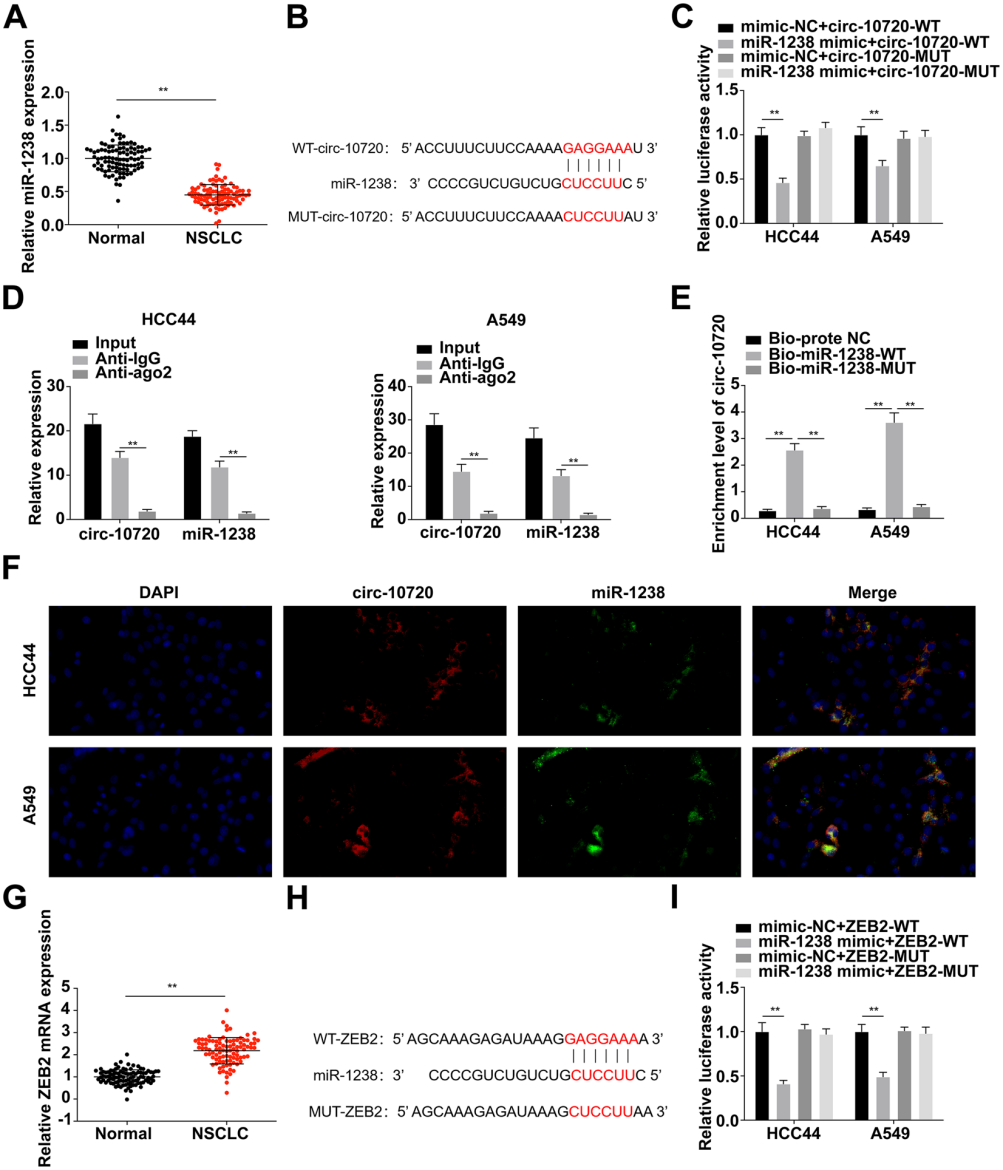
Circ-10720 modulates ZEB2 via sponge adsorption of miR-1238. **A** Detection of miR-1238 in NSCLC tissues and para-cancerous tissues by RT-qPCR; **B** prediction of the binding site of circ-10720 and miR-1238 by the bioinformatics website; **C** verification of binding of circ-10720 with miR-1238 by the dual luciferase; **D** test of endogenous association of miR-1238 with circ-10720 by RIP; **E** examination of the enrichment of miR-1238 to circ-10720 by RNA pull-down; **F** FISH showed that circ-10720 and miR-1238 were co-localized in the cytoplasm of A549 and HCC44 cells; red represents circ-10720 and green represents miR-1238; **G** detection of ZEB2 mRNA in NSCLC tissues and para-cancerous tissues by RT-qPCR; **H** prediction of the targeting of ZEB2 with miR-1238 by the bioinformatics website; **I** targeting of ZEB2 with miR-1238 detected by the dual luciferase reporter assay; *P < 0.05, **P < 0.01. *N* = 3; the data in the figure were in the form of mean ± SD

As shown in Fig. 4A, miR-1238 was downregulated in NSCLC tissues versus the para-cancerous tissues. The bioinformatics website miRanda and RNAhybrid cross-screened and found that circ-10720 and miR-1238 have targeted binding sites (Fig. 4B). The luciferase activity of the co-transfection with miR-1238 mimic and wild-type circ-10720 was reduced, while no distinct differences were seen in the luciferase activity of mutant 3’UTR versus the mimic-NC (Fig. 4C). Circ-10720 and miR-1238 in Anti-AGO2 were abundant versus the Anti-IgG (Fig. 4D). As presented in Fig. 4E, the enrichment of circ-10720 in the Bio-miR-1238-WT was elevated, while the enrichment of circ-10720 in the Bio-miR-1238-MUT had no distinct differences versus the Bio-probe NC. Furthermore, FISH analysis revealed that circ-10720 and miR-1238 were co-expressed in the cytoplasm of A549 and HCC44 cells (Fig. 4F). Together, circ-10720 interacted with miR-1238.

Meanwhile, we further confirmed that ZEB2 mRNA was elevated in NSCLC tissues versus the para-cancerous tissues (Fig. 4G). MiR-1238 and ZEB2 shared targeting sites (Fig. 4H). Subsequently, a dual-luciferase reporter assay was carried out (Fig. 4I), which manifested that the luciferase activity of the cells co-transfected with wild-type ZEB2 and miR-1238 mimic was reduced, while the luciferase activity of the mutant ZEB2 and miR-1238 mimic was not changed.

Collectively, circ-10720 acted as a sponge of miR-1238 to modulate ZEB2.

### Knockdown of circ-10720 represses NSCLC tumor growth in vivo

**Fig. 5:**
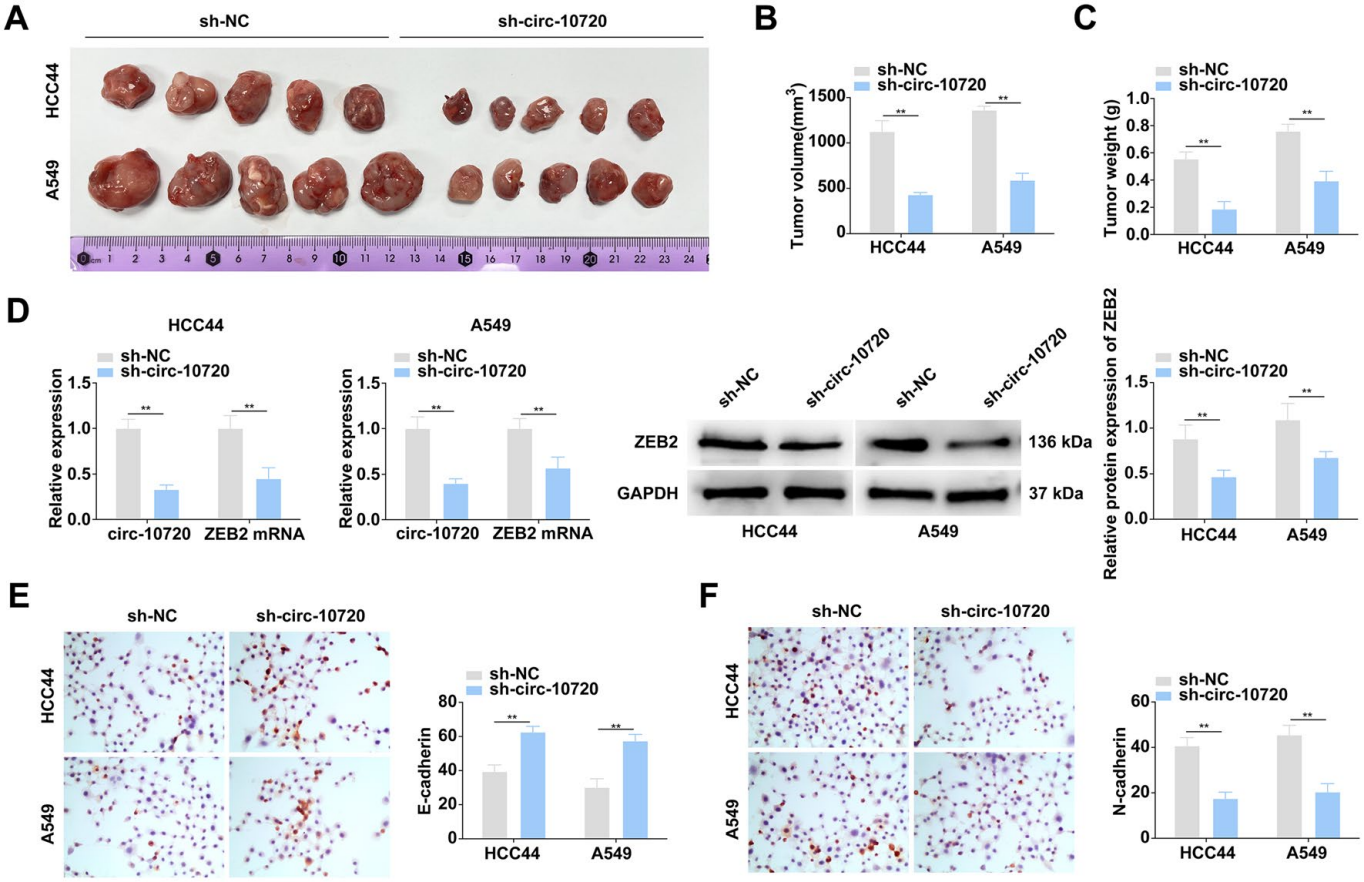
Knockdown of circ-10720 represses NSCLC tumor growth in vivo. **A** Images of xenograft tumors; **B** result of xenograft tumor volumes; **C** result of xenograft tumor weights; **D** relative expression of circ-10720, miR-1238, and ZEB2 in xenograft tumor tissues were measured by RT-qPCR and Western blot; **E** expression of E-cadherin in xenograft tumor tissues was evaluated by IHC; **F** expression of N-cadherin in xenograft tumor tissues was evaluated by IHC; *P < 0.05, **P < 0.01. *N* = 5; the data in the figure was in the form of mean ± SD

A mouse xenograft tumor model was conducted to figure out the impact of circ-10720 on NSCLC in vivo. The results manifested that knockdown of circ-10720 remarkably suppressed the tumor volume and weight of mice (Fig. 5A–C). Circ-10720 and ZEB2 expression levels in tumors were verified by RT-qPCR and Western blot. It was revealed that circ-10720 and ZEB2 were decreased in the sh-circ-10720 group (Fig. 5D).

Detection of E-cadherin and N-cadherin in the transplanted tumor tissue of nude mice was performed using immunohistochemical staining (Fig. 5E, F). The expression of N-cadherin was lower, whereas that of E-cadherin was higher in the sh-circ-10720 group.

## Discussion

NSCLC ranks first in global cancer mortality, and tumor metastasis is the leading cause of death, but the underlying mechanisms have not been fully elucidated. CircRNAs were once incorrectly thought to be byproducts of splicing errors and to have no biological function [42]. However, increasing studies in recent decades have shown that circRNA participates in multiple biological processes. In addition, circRNAs in non-proliferating cells are more stable and more abundant than their corresponding linear RNAs compared to proliferating cells. CircRNA is usually generated by RNA splicing of protein-coding gene transcripts [43]. Evidence manifests that circRNA is aberrantly expressed in human cancers [44].

MiRNA is non-coding RNA that modulates post-transcriptional genes via repressing mRNA translation and reducing mRNA stability [45]. Studies have clarified that miRNAs exert biological functions primarily through modulating the downstream targets or vital factors in tumor cell signaling pathways [46]. In addition, some miRNAs are dysregulated in multiple human cancers including NSCLC, and some specific miRNAs are able to be adopted as potential biomarkers for cancer diagnosis and treatment. For instance, miR-221 exerts a carcinogenic function in NSCLC via directly targeting TIMP2 [47]. Moreover, a new regulatory mechanism has been discovered and widely studied in recent years, that is, circRNA is available to compete with miRNA as a ceRNA, leading to the loss of miRNA function [48].

EMT is the crux of cancer metastasis [49]. The EMT process is an essential component of development, wound healing, and stem cell behavior. In addition, it plays a crucial role in promoting pathologic fibrosis and cancer progression [50]. EMT in the early progression of cancer metastasis can weaken cell adhesion, and enhance cell migration [51]. CircRNA is recognized as an efficient and specific regulator of EMT. For example, Ren et al. reported that circ_0043265 represses the EMT process of NSCLC via the miR-25-3p/FOXP2 pathway [52].

In our study, we showed that circ-10720 was elevated in NSCLC tissues and cells, and its expression level was correlated with TNM stage of patients. Our findings are consistent with previous studies that circRNAs are aberrantly expressed in NSCLC and correlate with clinicopathologic features of NSCLC patients. For instance, an investigation by Zhang et al. demonstrated that circ_0014130 is elevated in NSCLC tumor tissues and is associated with TNM staging and metastasis [53].

Furthermore, we clarified that knockdown of circ-10720 elevated E-cadherin protein, repressed N-cadherin and vimentin expression. In addition, in a mouse model experiment, circ-10720 deficiency-triggered tumor cell growth and EMT inhibition were demonstrated. It is well acknowledged that circRNA modulates cancer progression as a ceRNA of miRNA. To illustrate, a recent study reported that circ_0020123, as a ceRNA of miR-488-3p, modulates ADAM9 to boost the progression of NSCLC [54]. In this study, miR-1238 was found to augment after circ-10720 knockdown. circ-10720 is supposed to be a ceRNA of miR-1238.

We further revealed that miR-1238 was silenced in NSCLC and repression of miR-1238 effectively turned around the influence of circ-10720 knockdown on the biology of NSCLC cells. In short, circ-10720-modulated NSCLC cell proliferation, migration, invasion, and EMT process via controlling miR-1238.

Finally, the downstream target gene (ZEB2) of miR-1238 was analyzed. The Zeb family consists of Zeb1 and Zeb2, both have N-terminal and C-terminal zinc fingers that bind to regulatory DNA sequences in their target promoters, which allows the Zeb family to participate in different biological events, such as embryogenesis, hematopoiesis, and EMT. ZEB2 is a transcription factor that drives EMT, and its expression is usually followed by Snail expression activation [55]. ZEB2 is highly expressed at the invasion site of GBC and improves the invasion potential by inhibiting the expression of E-cadherin and T-cadherin and increasing the expression of N-cadherin and Vimentin at the transcriptional level [56]. Previous studies have indicated that ZEB2 induces EMT by inhibiting the expression of E-cadherin, promotes cell migration and invasion, and plays a pro-cancer role in many types of cancer, such as breast cancer [57], hepatocellular carcinoma [58] and NSCLC [59, 60]. In our study, we designed and conducted rescue experiments and showed that overexpression of ZEB2 can partially reverse the decrease of N-cadherin and Vimentin expression caused by overexpression of miR-1238, as well as the increase of E-cadherin expression, suggesting that ZEB2 induces EMT by inhibiting the expression of E-cadherin and promoting the expression of N-cadherin and Vimentin in NSCLC. ZEB2 may be involved in promoting the development of NSCLC by rescuing the tumor inhibition effect of miR-1238.

## Conclusion

In brief, our study provides evidence that down-regulation of circ-10720-modulated ZEB2 by performing as a sponge of miR-1238 to suppress the proliferation, migration, invasion, and EMT as well as advance apoptosis of NSCLC cells. These findings provide new insight into a novel target therapy for NSCLC.

## Data Availability

The data sets used and/or analyzed during the present study are available from the corresponding author on reasonable request.
